# Maternal and Early-Life Circadian Disruption Have Long-Lasting Negative Consequences on Offspring Development and Adult Behavior in Mice

**DOI:** 10.1038/s41598-017-03406-4

**Published:** 2017-06-12

**Authors:** Benjamin L. Smarr, Azure D. Grant, Luz Perez, Irving Zucker, Lance J. Kriegsfeld

**Affiliations:** 1 Department of Psychology, University of California, Berkeley, CA USA; 2 Department of Integrative Biology, University of California, Berkeley, CA USA; 3 The Helen Wills Neuroscience Institute, University of California, Berkeley, CA USA

## Abstract

Modern life involves chronic circadian disruption through artificial light and these disruptions are associated with numerous mental and physical health maladies. Because the developing nervous system is particularly vulnerable to perturbation, we hypothesized that early-life circadian disruption would negatively impact offspring development and adult function. Pregnant mice were subjected to chronic circadian disruption from the time of uterine implantation through weaning. To dissociate *in utero* from postnatal effects, a subset of litters was cross-fostered at birth from disrupted dams to control dams and vice versa. Postnatal circadian disruption was associated with reduced adult body mass, social avoidance, and hyperactivity. *In utero* disruption resulted in more pronounced social avoidance and hyperactivity, phenotypes not abrogated by cross-fostering to control mothers. To examine whether circadian disruption affects development by acting as an early life stressor, we examined birthweight, litter size, maternal cannibalism, and epigenetic modifications. None of these variables differed between control and disrupted dams, or resembled patterns seen following early-life stress. Our findings indicate that developmental chronic circadian disruption permanently affects somatic and behavioral development in a stage-of-life-dependent manner, independent of early life stress mechanisms, underscoring the importance of temporal structure during development, both *in utero* and early postnatal life.

## Introduction

Organisms across taxa exhibit daily rhythms in behavior and physiology generated by endogenous ~24 h oscillations (circadian rhythms) that facilitate synchronization to the day-night cycle^1^. Circadian oscillations are ubiquitous in cells throughout the brain and body and their coordinated daily activity is essential for normal health^1–3^. Disruption to the 24-hour light cycle desynchronizes rhythmic systems throughout the brain and periphery, dysregulating physiological networks and inducing suboptimal physiological functioning until systems realign. When these disruptions are acute (e.g., in the case of a single ‘jet lag’), adults can achieve internal realignment with limited negative impact to health^4^. However, when circadian disruptions are chronic, as in shift work or frequent travel across time zones, long term physiological and neurological health deteriorate^2, 5^. In modern settings, work and school schedules, artificial light, and portable electronic devices have made chronic circadian disruptions a near-universal part of the landscape.

In adult mammals, chronic circadian disruption has diverse negative impacts on physical and mental health^6, 7^, including a shortened lifespan^8^. Because developing organisms are sensitive to environmental perturbations^9–12^, we speculated that developmental chronic circadian disruption (DCCD) would negatively impact normal development, with consequences persisting into adulthood. Furthermore, because different systems develop at different ontogenetic stages, we hypothesized that the timing of DCCD would differentially impact distinct physiological and behavioral traits. In previous studies, exposing pregnant mice and rats to constant light produced lasting increases in anxiety-like behavior^13–15^, with mixed results in learning tasks^14–16^ and associated hippocampal functions^15, 16^. Likewise, maternal circadian disruption alters offspring metabolic functioning in adulthood^13, 17–19^, including the regulation of glucocorticoids^17^ and insulin^18^.

Whether these outcomes are a consequence of circadian disruptions *in utero* or from alterations in maternal care by circadian-disrupted mothers has not been disambiguated. Thus, the first goal of this investigation was to determine the contributions of circadian disruptions at different developmental life stages to adult deficits. In addressing this question, we elected to employ manipulations that would mimic the type of disruptions – phase shifts – experienced by pregnant women under rotating shift schedules. We therefore implemented a repeated phase-shift design rather than exposure to constant light or only a single “jet lag”. To dissociate the impact of circadian disruption *in utero* from changes in maternal care resulting from circadian disruption, DCCD mice were cross-fostered to control mothers at birth. To isolate the impact of DCCD to the postnatal period, control animals were cross-fostered to DCCD mothers.

The second goal was to investigate putative mechanisms for the observed effects. Previous studies have proposed that perturbed maternal melatonin signaling is a mechanism by which circadian disruption negatively impacts development^15, 17, 20–25^. For instance, hippocampal clock gene rhythms are disrupted after gestation in constant light, and timed infusions of melatonin delivered to the dam prevent these deficits in adult offspring^15^. In the present study, we used BALB/c mice that have markedly attenuated melatonin production^26, 27^ to eliminate any effects of circadian disruption resulting from dysregulated melatonin.

Because chronic circadian disruption can activate the stress axis^28^, we examined whether or not adverse effects of DCCD are associated with well-established markers of early life stress (ELS). ELS, often transmitted to offspring through dams’ poor maternal care, has lasting developmental effects that vary depending on the life stage in which the stress is experienced^29^. ELS results in hippocampal hypermethylation at the promoter regions of genes related to the stress axis and brain development, including glucocorticoid receptor (NR3c1) region 1_7_^29–31^ and brain derived neurotrophic factor (BDNF) region IV^32–34^. When disruptions occur *in utero*, this epigenotype is rescued by cross-fostering pups to an unstressed dam^29^. Along with hypermethylation of stress-related genes, maternal stress and ELS are associated with decreases in birth weight^35, 36^ and litter size^37^, and increases in maternal cannibalism of pups^38, 39^. If DCCD impacts offspring by acting as a maternal stressor, then disrupted dams should show reduced maternal care and DCCD offspring should exhibit methylation patterns seen in offspring born to stressed dams.

## Methods

### Animals

**Figure 1:**
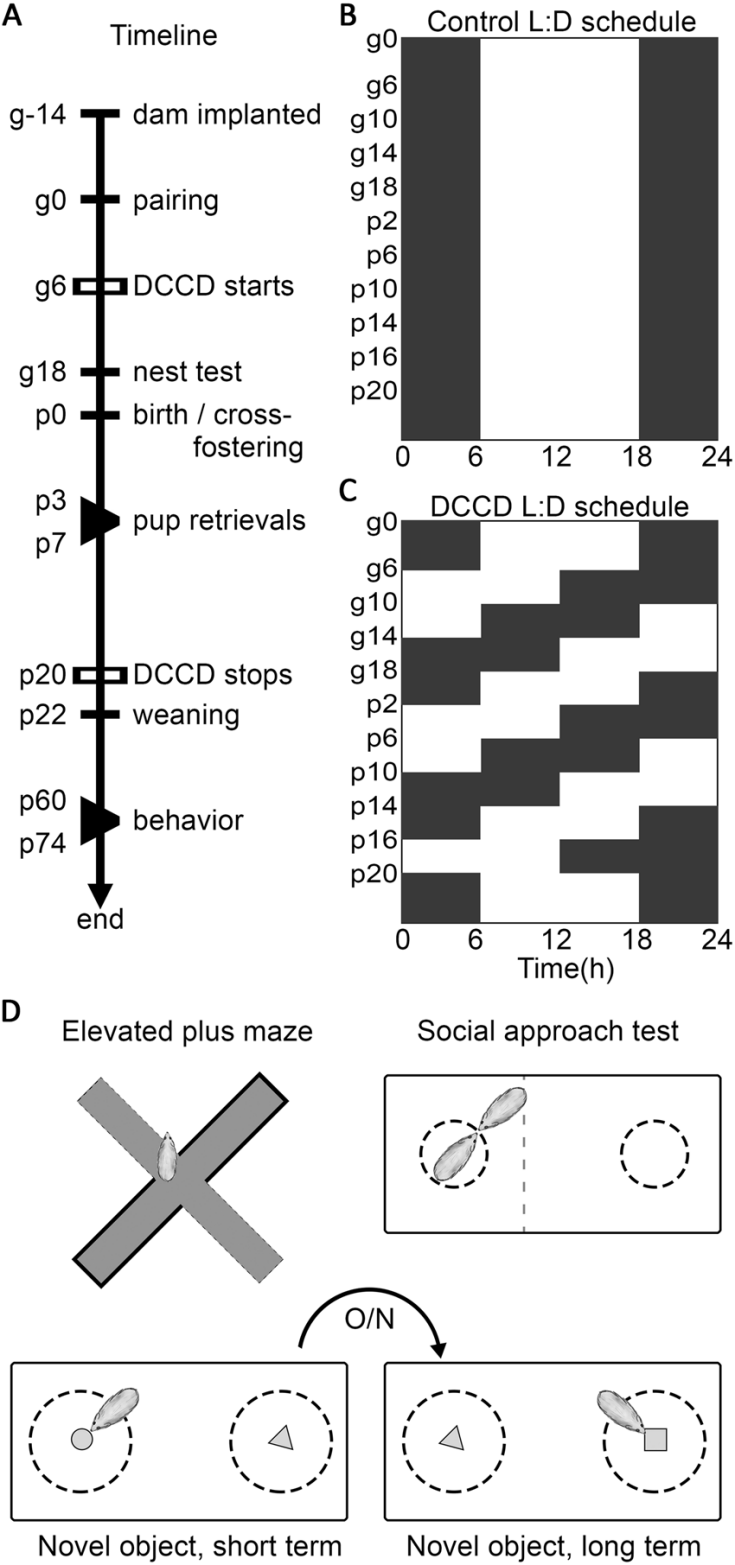
Experimental outline. Timeline (**A**) of experiment from maternal sensor implantation through end point for adult offspring. LD schedule for control animals (**B**) and DCCD animals (**C**) during the experiment, with advances every four days from the time of uterine implantation through weaning. Cartoons of behavioral tests administered to adult offspring during the time labeled “behavior” on the timeline (**D**).

All procedures were approved by the UC Berkeley Institutional Animal Care and Use Committee (IACUCs) and are in accordance with NIH policies on the care and use of animals. Female BALB/c mice were mated between 2–3 months of age, with males 2–4 months of age. Mice were housed under an LD 12:12 photocycle with *ad libitum* access to water and laboratory chow. Light onset and offset occurred at 0600 h and 1800 h, respectively. Light intensity during the photo- and scotophases were ~400 lux white light and <1 lux red light, respectively. Humidity and temperature were held constant at 40% and 21 °C, respectively. Mice were group-housed until two weeks before first pairings; thereafter males and females were singly housed. Two weeks before pairing Mini Mitter G2 E-Mitter transmitters (Starr Life Sciences Co., Oakmont, PA) were implanted in the intraperitoneal cavity under isoflurane anesthesia, followed by subcutaneous injections of 0.03 mg/kg buprenorphine in saline, administered every 12 h for 2 days after surgery. The transmitters were sutured to the ventral muscle wall to maintain consistent measurements and chronically recorded locomotor activity (LA) and core body temperature (CBT). The timeline of experimental procedures is presented in Fig. 1. A subset of Mini Mitter temperature and activity data from dams were communicated in a previous report^40^. All experiments were carried out in compliance with the NIH guide for the care and use of laboratory animals and in accordance with protocols approved by the Animal Care and Use Committee at The University of California, Berkeley.

### Housing and Circadian Disruption

CBT profiles were utilized to monitor estrous cycles; mice were paired for 17 h on the day of estrus from 2 h before light offset (1600 h) to 3 h after light onset the following day (0900 h). A male was introduced to the female’s home cage, permitting specification of the day pregnancy was initiated. Pregnancies were confirmed by monitoring the daily CBT profile^40^. After each round of pairing, half of the females were moved to a separate light box and, on gestational day 6 (g6 – one day after uterine implantation) were exposed to DCCD consisting of 6 h advances in the light cycle every 4 days. DCCD continued until weaning at 22 days of age, at which point offspring were returned to a stable 12:12 LD cycle for the remainder of the study. The other half of the females remained in the static 12:12 LD cycle for the duration of the study. For cross-fostered litters, pups were switched with another litter within 24 h of birth. Six groups of litters were generated: “controls” (C) were neither shifted nor cross fostered; “shifted” (S) animals were exposed to DCCD but not cross-fostered; and all four permutations of control and shifted litters were generated (CC, CS, SC, SS, where the first letter denotes the condition *in utero*, and the second the condition *post-partum*). All mice were weighed at birth, daily from day postnatal day (p) 3-p7, at weaning (p22), midlife (p33), and in adulthood (p60). C and S offspring were weighed every 2–3 days after weaning until p60. The date of first vaginal opening was recorded. At weaning mice were ear-punched for identification and separated by sex within litters to a maximum of 4/cage. In litters with 5 same-sex siblings, animals were separated into groups of 3 and 2 to avoid isolation; offspring with litters of just 1 same-sex sibling were singly housed.

### Examination of Maternal Behavior

Nest building was assessed prior to parturition, and pup retrieval during lactation. To measure nest building, nest material was removed from the cage two hours before dark onset two days before expected parturition^40^; pre-weighed fresh nesting material was placed on top of the cage lid, where it could be pulled down into the cage by the pregnant mouse. Four hours after the following light onset (18 h later), the nest material remaining on the cage lid, if any, was weighed. The nest was assessed for doming, consolidation, and neatness. Nests were scored from 1–4, with 1 representing no nest and 4 a complete, consolidated, domed nest. Nest building was only assessed in C and S groups, as it preceded cross-fostering.

To further examine maternal care, a pup retrieval test was conducted. Three hours after light onset on p3, the dam was placed into a 6 × 6′ container and pups were weighed and maintained under a warming lamp. After 5 min, 4 pups chosen at random were returned to the side of the home cage farthest from the nest. The dam was then placed on the nest and the time for each pup to be retrieved to the nest was recorded. This procedure was repeated daily from p3 through p7.

### Offspring Behavioral Assessment

Beginning in adulthood, mice began a regimen of one behavioral test/day. In sequence, testing consisted of the elevated plus maze^41^, social approach behavior^42^, short-term novel object recognition (within day), and long-term novel object recognition (across days)^43^. The tests selected allowed assessment of exploration and anxiety-like behavior, social behavior, and learning, while minimizing both time spent in the apparatus and associated stress. These considerations were intended to minimize the impact of earlier tests on subsequent examination. The order of testing allowed novel arena exploration in the elevated plus maze without confounds of previous learning experience or possible stress from social encounters. Learning was assessed last as it required the longest duration of time away from the home cage, and potential anxiety in subsequent tests. All behavioral assessments were carried out in the middle six hours of the light phase. Mice were given 1 h to acclimate after being moved from their housing room to the testing room, after which they were moved through the assessments in an order blind to sex and condition. A video camera was suspended above the center of the testing areas and all relevant behaviors recorded for scoring in vCode, validated by at least two independent scorers.

#### Elevated plus maze

The mazes were constructed in house from ¼″ acrylic, laser-cut into four arms to fit together at 90° angles with a 2″ wide, 8″ long floor, two of which were enclosed on three sides to a height of 6″, joined in the center by a 2″ × 2″ square, and painted matte black. For testing, the maze was elevated 3’ off the ground on a tripod. Mice were placed in the center of the apparatus and allowed 4 minutes of exploration^41^. After each mouse completed the test, all fecal pellets were removed, the arena sprayed with ethanol, wiped with paper towels, and allowed to dry before the next mouse was tested. Mice were scored for % time in open vs. closed arms, for stereotyped anxiety-like movements^41^, taken as the sum of arm switches, rearing, full-body extension, and dipping of the head over the edge of the open arms.

#### Social approach arena

Two 3″ diameter cylindrical cages were set with centers 12″ apart in a 10″ × 18″ housing chamber with ¼″ bedding. The cages were centered relative to the walls of the chamber so that a mouse could move completely around each cage. Cages were constructed of clear plastic, with vertical ¼″ × 2″ slits every ½″ of the circumference so that mice could touch noses through the slits, but not move through the slits. One cage housed an age and sex-matched “stranger”, the other cage was empty, with the sides randomized on each trial (modified from ref. 42). Focal mice were placed into the center of the arena and given 1 min for familiarization. The subsequent 3 min were scored for % time the mouse moved more than 1″ toward the mouse-containing cage from the midline of the arena.

#### Novel object recognition, Day 1 (NOR1)

Mice went through three stages of novel object recognition (NOR) testing on day 1: For habituation, mice were placed in an empty 10″ × 18″ animal housing chamber. For familiarization, animals were placed in the same arena with 2 identical objects separated by 12″, centered with respect to the shorter arena dimension. For novel object recognition, one of the familiar objects was replaced with a novel object of similar size and complexity. Objects consisted of a 1.5″ wing nut, a 1.5″ bolt, or the wing nut fitted to the end of the bolt. Side placement and order of exposure were randomized. Each mouse was tested for 4 min per stage, and scored for time per half (for habituation), time attending to each object (scored when the object was within the front 90° of the mouse’s head and within one body length of the mouse’s head), and time touching each object with its face or paw. A 30–60 min interval separated testing between the stages^43^.

#### Novel object recognition, Day 2 (NOR2)

This test consisted of a single stage on the day following NOR1. The arena was arranged as for novel object exploration on day 1, but the novel object was different than the object used in day 1. Replacement side was randomized as was order of object introduction across days.

### Examination of GR and BDNF Gene Methylation

Hippocampi were dissected from bisected snap-frozen brains. DNA was extracted using a protocol modified from the DNEasy Blood and Tissue kit (Qiagen, Hilden Germany, product # 69504), wherein samples were incubated with proteinase K overnight. Extracted DNA was quantified using a Nanodrop 1000 kit (Thermo Fischer Scientific, Waltham, MA) and 500 ng-1 ug of DNA were bisulfite converted using a Qiagen Epitect Bisulfite Kit (product # 59104) according to the manufacturer’s instructions. Methylation occurs largely at cytosines followed by guanines (termed CpG sites). Bisulfite treatment selectively replaces un-methylated cytosines with uracil, allowing methylated and un-methylated sites to be distinguished by Sanger Sequencing.

Bisulfite specific primers were designed using MethPrimer (Li Lab, Chinese Academy for Medical Sciences, Peking, China) and Bisulfite Primer Seeker (Zymo, Irvine, CA). Regions of BDNF and GR were chosen based on a consistent literature showing hypermethylation of the region 4 promoter^32, 33, 44^ and exon 1^7^ promoter^30, 31, 44, 45^ respectively. PCR was carried out using a ZymoTaq HotStart Polymerase kit as follows: 10 min at 95 °C denaturation, followed by 35 cycles of 30 s at 95 °C, 30 s at annealing temperature, 30 s at 72 °C, and a 7 min 72 °C final extension. Primer sequences used are listed in Table 1.

**Table 1:** Primer sequences for methylation screens.

*Gene*	*Region*	*Bisulfite Converted Primer Sequence*
NR3C1	Promotor 1_7_	F: GTTTATTTYGAGGTAGAGGAGGTATTATATG
		R: CCCCAAAATTCTAAACTCTTACTATATATTTCCCCTTC
BDNF	Promotor IV	F: GAGTTTTYGAGGGGGTGATAGTTAG
		R: TCCCCCCCCRAACTCACAATATATATAC

Primer bias, annealing temperatures, amplicon location, and CpG sites analyzed are presented in supplementary Fig. S1. PCR products were cleaned and sequenced by the Barker Sequencing Facility at University of California, Berkeley, using Axyprep MagPCR cleanup reagent according to the manufacturer’s instructions. All samples were bisulfite-converted and sequenced in duplicate.

Methylation was quantified as the nucleotide bases’ C/(C + T) peak height ratio as previously described^46^. Only samples with a conversion efficiency >88% were included in the analysis. Responders and non-responders were chosen based on the severity of sociality and weight phenotypes, (i.e., lowest weight and least social vs highest weight and most social for each group). Both sexes were examined, and no sex differences were apparent. For comparisons to the literature values of fold-change in methylation induced by early life stress, fold-changes in methylation were extracted from studies examining amplicons within GR 1_7_^29, 30, 47^ and BDNF IV^32–34^. The mean fold change between control and treatment groups across studies for each region was applied to our control rates to determine predicted methylation levels from our samples had they been obtained under early-life stress conditions.

### Statistical Analysis

Statistical analysis was carried out in Matlab 2016a. Weight progression, methylation state, and all behaviors were assessed by Wilcoxon rank-sum non-parametric comparisons (reported with *p*-value only) or Kruskal-Wallis non-parametric tests for group effect (reported with group statistic and *p-*value), as appropriate. Post hoc tests were carried out with Dunn’s comparisons, with alpha value set to *p* < 0.05, reported only as above or below threshold. N’s for each condition were as follows for females and males respectively: C: 19, 20; CC: 44, 39; CS: 22, 21; SC: 19, 16; SS: 18, 21; S: 24, 21. Maternal behavior N’s were as follows: C: 10; CC: 11; CS: 7; SC: 7; SS: 8; S: 11. Clusters were constructed using kernel based distribution inference for each group, and visualized with contours over the projected surfaces at the level of 50% inclusion and 0% inclusion (the surface apex, which defines the cluster centroid). Cluster dissimilarity was calculated with a Euclidean distance technique^48^, using a Kruskal-Wallis test for effect of group on the distances of all C cluster points with its centroid versus the distances from all points in each other cluster to the C centroid. Figures were produced in Matlab 2016a and formatted in Adobe Photoshop CS5.

## Results

### Maternal care is compromised by the light environment and *in utero* condition of offspring

**Figure 2:**
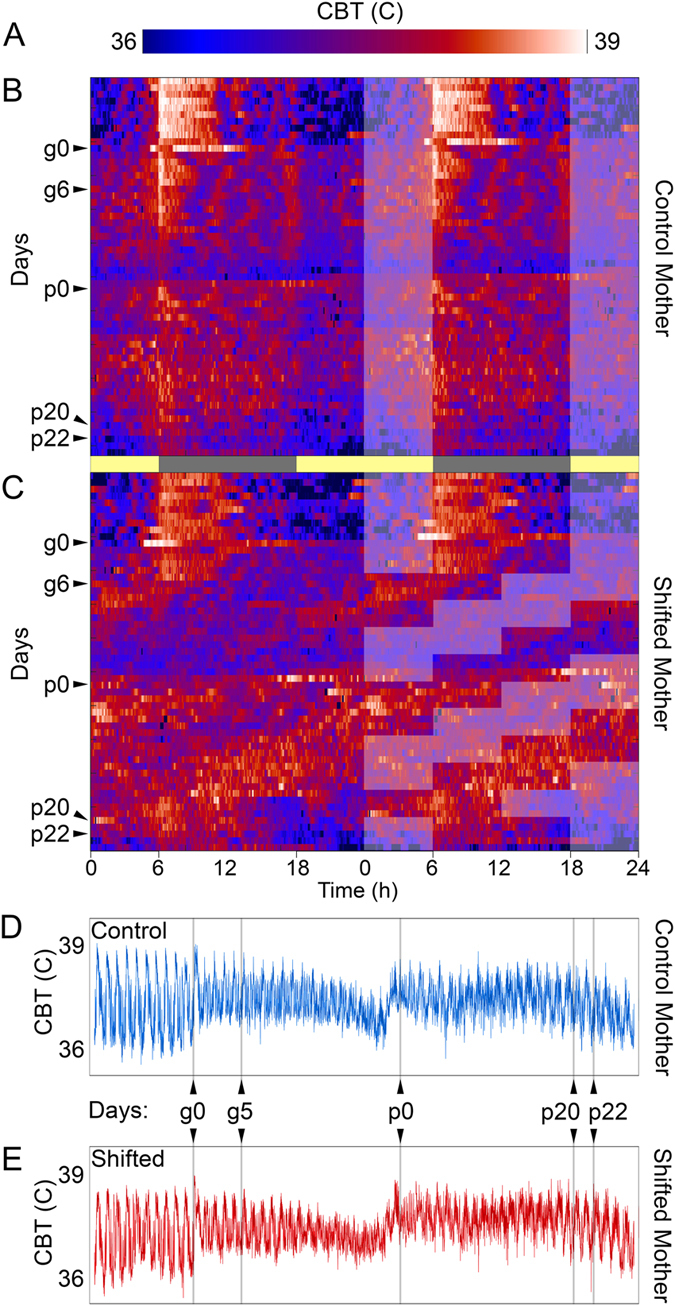
DCCD impacts maternal physiological rhythms. Color maps of intensity (**A**) reveal stable daily rhythms core body temperature (CBT) in sensor-implanted dams from control (**B**) and shifting (**C**) dams before onset of DCCD. Double-plotted actograms, with light-phase overlaid on the right half. After onset of DCCD, shifting dams show expected advances in their CBT rhythms in response to advances, which overall give shifting dams 2 additional daily cycles during their pregnancies (48 h accumulated advance). Nevertheless, pregnancies are of the same duration for control (**D**) and shifting (**E**) dams, as revealed by the stereotyped profile of CBT changes across pregnancy.

For dams exposed to repeated 6 h phase advances, physiological rhythms and locomotor activity showed chronic misalignment as females attempted to realign to the shifting LD cycle. This disruption is apparent in CBT (Fig. 2a,b) and LA (Supplemental Fig. 2a,b) raster plots. Despite the fact that the DCCD paradigm exposes dams to 2 additional subjective days during their pregnancy (48 h accumulated shifting), the number of calendar days for completion of pregnancy, as revealed by CBT, remains the same as for control mice (Fig. 2c,d).

**Figure 3:**
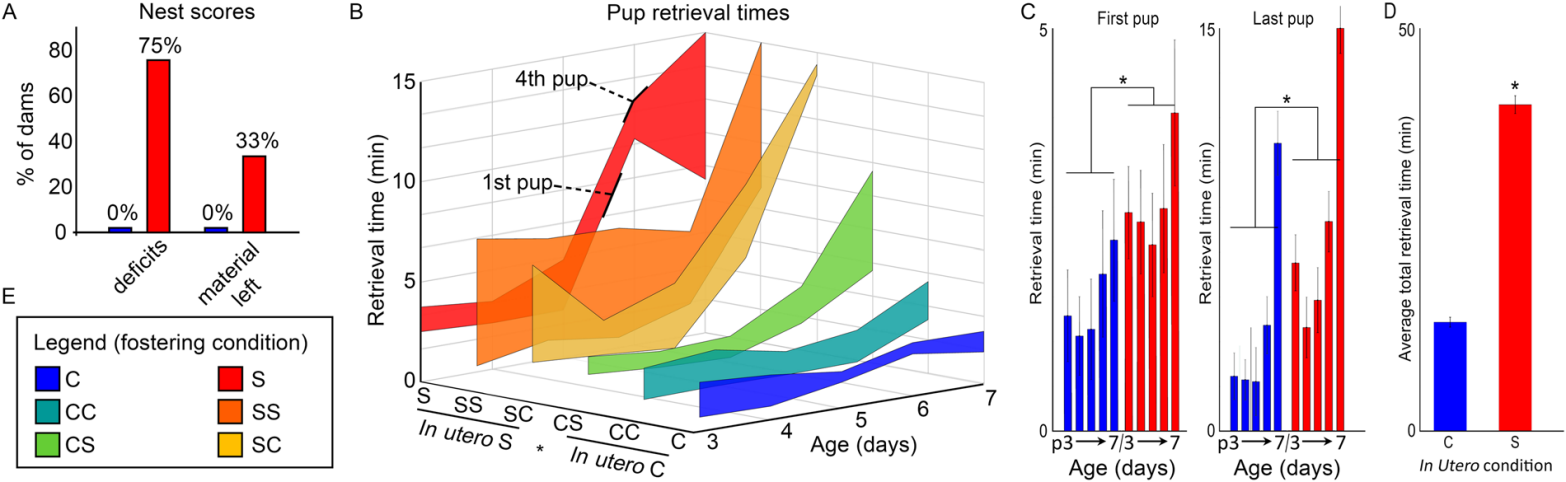
Maternal Behavior is altered by prenatal and postnatal DCCD. Before giving birth, 75% of S dams create nests with major structural deficits, and 33% do not use all nest material provided over the night of testing. By contrast, 0% of C dams exhibit these deficits in nest building (**A**). The condition of the pup *in utero* affects maternal pup retrieval behavior (**B**–**D**). A 3-dimenstional plot showing the time interval from retrieval of 1st to 4th pup (labeled as such on S data), which defines a region shaded by condition (B, legend in E) across 5 sequential days of retrieval trials. Pups experiencing *in utero* disruption (S, SS, SC) show longer retrieval times than pups from *in utero* control conditions (C, CC, CS), regardless of rearing-dam’s condition. As a group, dams retrieving pups that experienced *in utero* disruption take longer to retrieve the first (C, left) and last (C, right) pup, and this effect is present across all five trial days. A significant effect is also found in total amount of time spent retrieving pups across all 5 days (**D**), with *in utero*-disrupted pups taking longer to be retrieved than pups without *in utero* disruption.

Maternal care was negatively affected by light environment and *in utero* condition of offspring. Whereas all C animals built complete nests and used all nesting material, 75% of S dams built incomplete nests (*p* = 9.3 × 10^−3^) and 33% did not use all the nesting material provided (*p* = 0.3, not significant) (Fig. 3a). After birth, pup retrieval times were also significantly increased by DCCD (χ^2^ = 20.85, *p* = 9 × 10^−4^) (Fig. 3b,c). Specifically, the *in utero* condition of the pups predicted the mother’s retrieval scores (Fig. 3b). Dams rearing pups that were shifted *in utero* took significantly longer to retrieve both the first pup (χ^2^ = 6.37, *p* = 0.01) and last pup (χ^2^ = 12.78, *p* = 4 × 10^−4^), regardless of maternal shifting history (Fig. 3c). Shifted mothers cross fostering pups from control mothers (SC dams) did not differ from SS and S dams on the first day of retrieval testing (*p* = 0.49), but took significantly longer than C and CC (*p* = 0.047). By contrast, CS dams took longer to retrieve pups than C and CC (*p* = 0.02), but did not differ from S and SS (*p* = 0.26). However, by day 5, CS pup retrievals were not significantly different from those of either control groups (C, CC, p = 0.06) or shifted groups (S, SS, *p = *0.06). To summarize, pups that were shifted *in utero* start with a maternal retrieval disadvantage that progressively worsens over days, regardless of the postnatal maternal condition. Pups from control mothers eventually experience longer retrieval times if exposed to DCCD early in postnatal life, indicating that S and SS pups are negatively impacted by both circadian disruption *in utero* and poorer maternal care in early life.

### Postnatal disruption and poor maternal care lead to growth deficits but do not alter puberty onset

**Figure 4:**
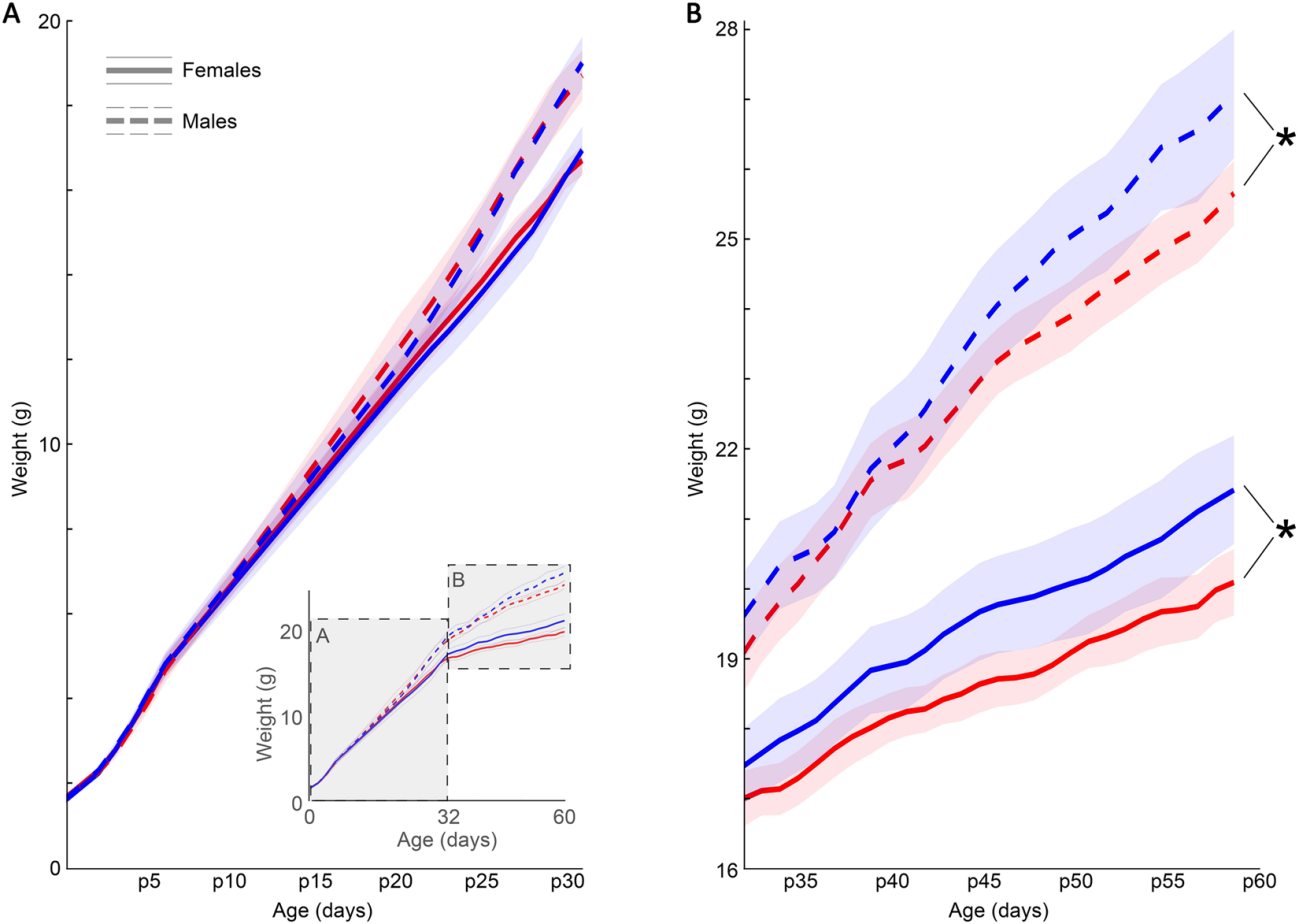
DCCD negatively affects offspring morphological development. Male and female offspring do not differ in body mass half-way through postnatal development (**A**), but by onset of adulthood (p60) S (red), offspring are significantly underweight in both males (dashed lines) and females (solid lines). Life-time growth curve show in (A, inset).

For C and S offspring, the week before midlife and the last week of maturation were chosen to assess adult growth. As expected, females weighed less than males regardless of condition both at p33 (*p* = 2.4 × 10^−4^) and in adulthood (*p* = 7.0 × 10^−11^). DCCD did not induce significant differences in body weight by p33 for either sex (females: *p* = 0.59, males: *p* = 0.53), but did cause lower adult weights in both females and males (*p* = 2.1 × 10^−3^, *p* = 2.2 × 10^−3^, respectively) (Fig. 4). Cross-fostered offspring were weighed only at p60 to assess adult weight (Table 2). An effect of group was also observed in cross-fostered litters (χ^2^ = 20.12, *p* = 0.0012 for males, χ^2^ = 11.11, *p* = 0.049 for females). In these cases, shifts during rearing more consistently lead to lower adult body mass than shifts *in utero* (χ^2^ = 10.01, *p* = 0.0016 for males by rearing condition, χ^2^ = 4.63, *p* = 0.03 for males as C & CC vs. conditions with shifting; χ^2^ = 6.3, *p* = 0.01 for females by rearing condition; χ^2^ = 2.1, *p* = 0.15 for females as C & CC vs. conditions with shifting). No difference was found in the date of first vaginal opening (mean ^+^/_−_ s.d., C: day 38 ^+^/_−_ 6; S: day 37 ^+^/_−_ 4).

**Table 2:** Body mass at p60, mean and standard deviation.

	*Sex*	*C*	*CC*	*CS*	*SC*	*SS*	*S*
*p60 Mass*	Female	21.3 ^+^/_−_ 1.9	20.4 ^+^/_−_ 2.0	20.5 ^+^/_−_ 2.5	21.1 ^+^/_−_ 2.1	20.1 ^+^/_−_ 2.5	19.8 ^+^/_−_ 1.4
	Male	26.8 ^+^/_−_ 1.6	25.0 ^+^/_−_ 2.7	24.4 ^+^/_−_ 1.7	26.0 ^+^/_−_ 1.9	24.5 ^+^/_−_ 2.2	25.5 ^+^/_−_ 1.5

### Early life circadian disruption leads to adult deficits in social and anxiety behaviors

**Figure 5:**
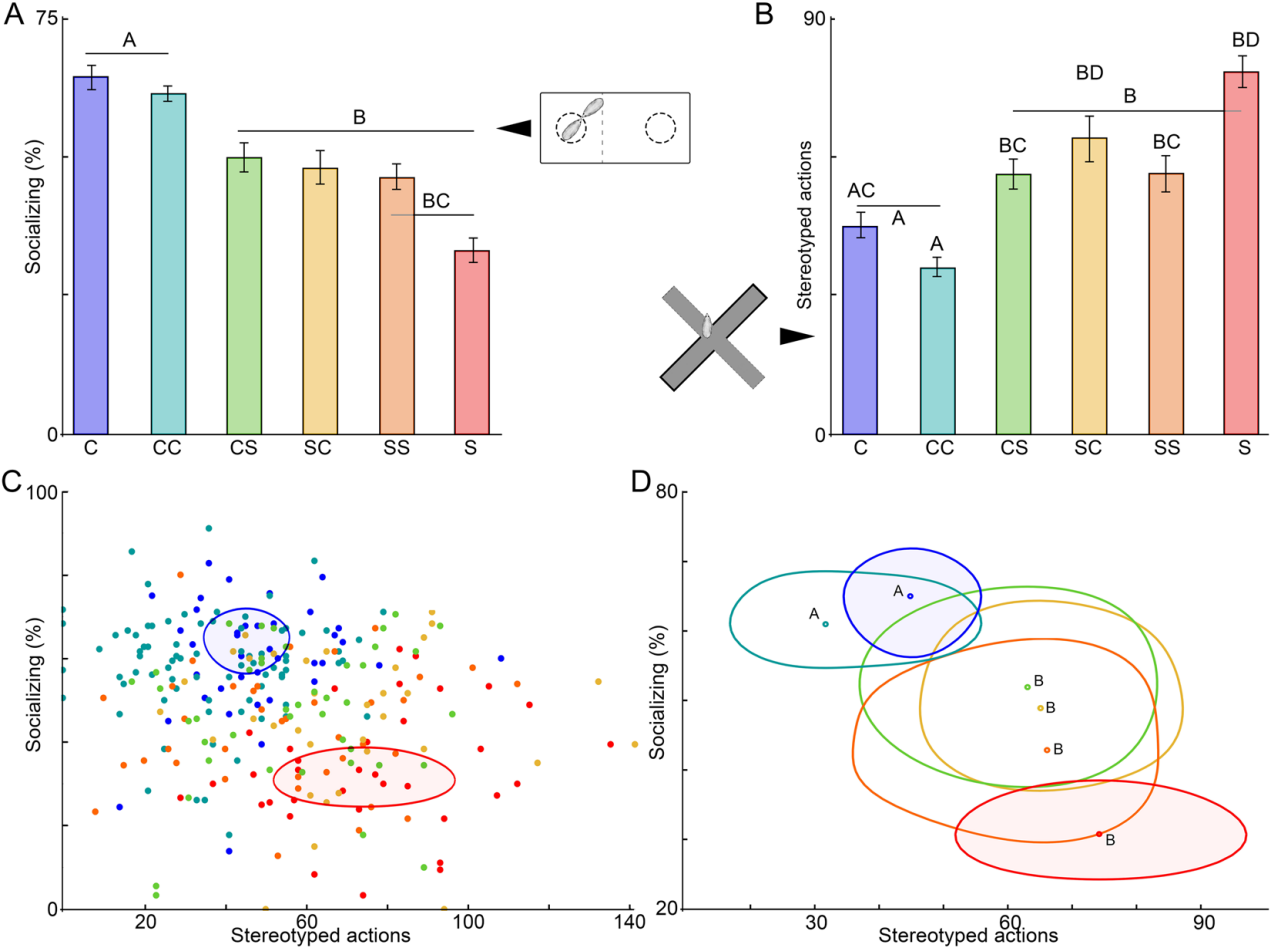
DCCD induces adult behavioral abnormalities. Mice exposed to DCCD exhibit a significant decrease in % time spent exploring a same-sex, same-age unfamiliar mouse (median ^+^/_−_ standard error) (**A**). DCCD offspring are hyperactive when exploring a novel elevated plus maze (median ^+^/_−_ standard error) (**B**). Note that SC offspring exhibit significantly greater separation from C and CC than do CS offspring. Individual socializing and hyperactivity scores plotted against each other reveal phenotypic separation by condition (**C**). Contours describing the 50% inclusion level for S and C are shaded. Contours for all groups (**D**) illustrate the significant separation of DCCD phenotypes from C phenotype. Groups with significant differences indicated by letters on bars; colors are consistent for groups across all plots.

In the Social Approach task, shifted offspring (S, SS, SC, and CS) spent significantly less time near the novel mouse than control offspring (C and CC) (χ^2^ = 80.99, *p* = 2.89 × 10^−16^; Fig. 5a). Post hoc analysis reveals that CS offspring were significantly more social than S, but less than C or CC (*p* < 0.05). No sex effect is apparent (*p* > 0.05). For the elevated plus maze, no difference was found for time in open vs. closed arms (mean time in open/closed arms: mean ^+^/_−_ s.d., C: 0.08 ^+^/_−_ 0.1, S: 0.2 ^+^/_−_ 0.4). There was, however, a significant effect of group in the frequency of stereotyped anxiety-like movements while in the maze (χ^2^ = 87.35, *p* = 2.42 × 10^−17^; Fig. 5b). No effect of sex was observed (*p* > 0.05). *Post hoc* analysis revealed that, generally circadian disruption whether pre- or postnatal, was associated with increased anxiety-like activity; S, SC CS, and SS offspring exhibited significantly more anxiety-like movements than C or CC animals, although CS and SS animals were significantly less active than S mice (*p* < 0.05). Finally, during both days of novel object recognition, all groups attended to the novel object more than 50% of the trial, and no effect of group or sex reached significance (% time near novel object: mean ^+^/_−_ s.d., Day 1: C: 63 ^+^/_−_ 18, S: 63 ^+^/_−_ 25; Day 2: C: 73 ^+^/_−_ 11, S: 67 ^+^/_−_ 17, Table 3).

**Table 3:** Novel object recognition % attending novel object, mean and standard deviation.

*NOR*	*Sex*	*C*	*CC*	*CS*	*SC*	*SS*	*S*
Day 1	Female	62 ^+^/_−_ 20	56 ^+^/_−_ 26	50 ^+^/_−_ 31	57 ^+^/_−_ 30	69 ^+^/_−_ 28	73 ^+^/_−_ 9
Day 1	Male	65 ^+^/_−_ 15	56 ^+^/_−_ 30	53 ^+^/_−_ 28	60 ^+^/_−_ 28	58 ^+^/_−_ 32	50 ^+^/_−_ 32
Day 2	Female	72 ^+^/_−_ 13	51 ^+^/_−_ 28	48 ^+^/_−_ 31	51 ^+^/_−_ 30	67 ^+^/_−_ 31	63 ^+^/_−_ 17
Day 2	Male	74 ^+^/_−_ 9	38 ^+^/_−_ 28	64 ^+^/_−_ 34	50 ^+^/_−_ 26	48 ^+^/_−_ 27	73 ^+^/_−_ 15

### Combined behavioral phenotyping reveals novel effects of DCCD on adult behavior

Plotting elevated plus scores against social approach scores creates two-dimensional scatter plots for all individuals. Treatment groups cluster within this scatter, and comparison of distance between clusters revealed a separation of S and C populations, with cross-fostered groups exhibiting an intermediate phenotype (overlapping clusters, Fig. 5c). Cluster similarity tests reveal a significant effect of group on cluster separation (χ^2^ = 45.79, p = 1 × 10^−8^). Probing this effect with a *post hoc* analysis indicated that CC mice were not significantly separable from C mice, but CS, SC, SS, and S were all in significantly separate clusters from C mice (*p* < 0.05) (Fig. 5d), indicating that prenatal and postnatal shifting have additive, detrimental effects on adult behavior.

### DCCD Does Not Mimic Early Life Stress

**Figure 6:**
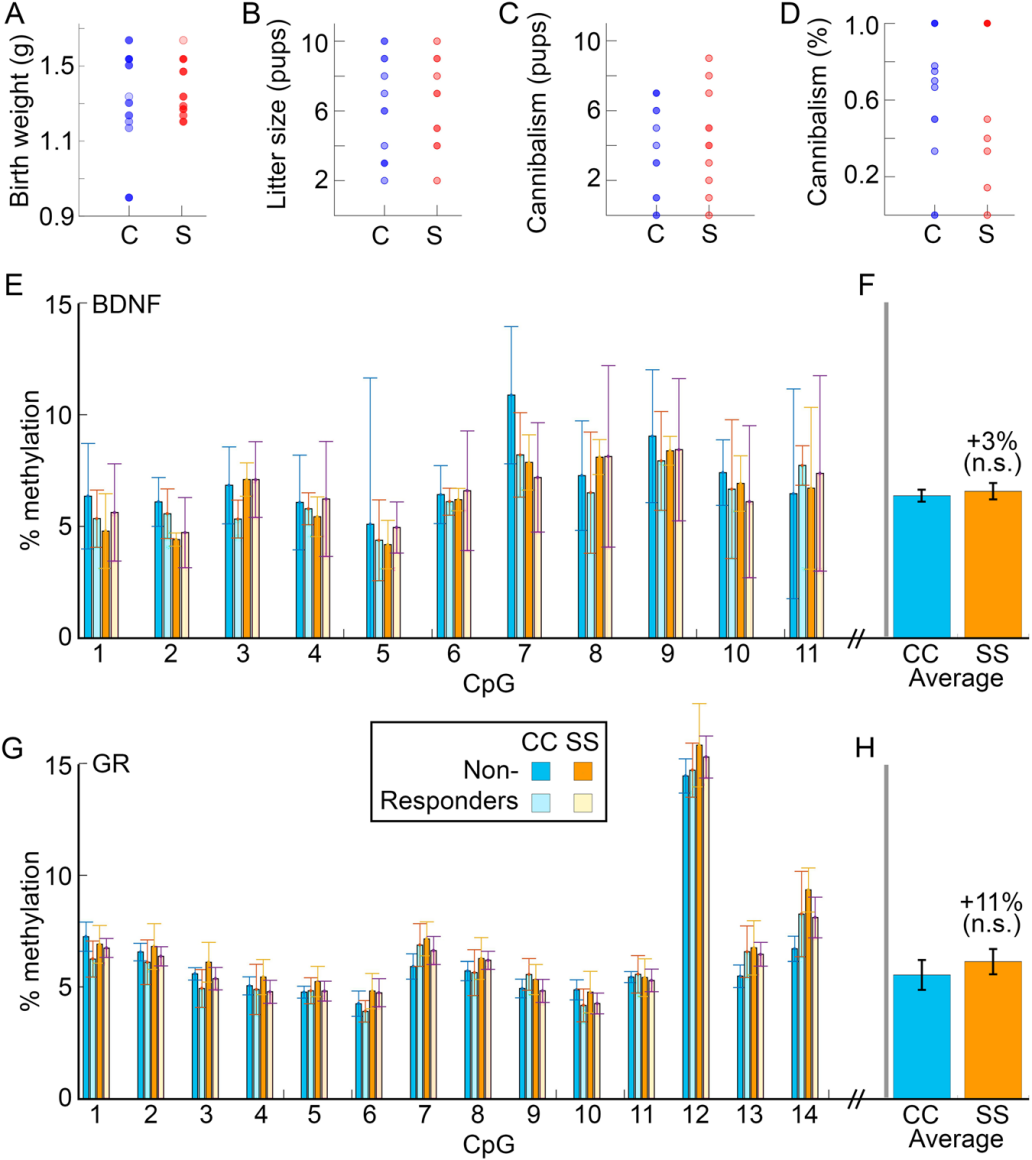
DCCD does not lead to differential methylation patterns of the GR and BDNF genes. Birthweight for S and C litters do not differ (**A**). Litter size (**B**), number of pups cannibalized by the mother (**C**), or percent of litters cannibalized (**D**) are not affected by circadian disruption. Hypermethylation of BDNF V (**E**) and GR_1_7 (**G**) regulatory regions of the genome is also absent in SS offspring. High and low responders were chosen from each group, and no apparent effect of phenotype on methylation rates.

Birth weight, number of pups per litter, number of pups cannibalized, and cannibalization rate did not differ between C and S dams (*p* = 0.80, *p* = 0.86, *p* = 0.70, and *p* = 0.69, respectively). SS and CC mice did not differ in methylation rate on the promotor regions of BDNF IV (χ^2^ = 1.12, *p* = 0.77) or GR 1_7_ (χ^2^ = 1.97, *p* = 0.58) (Fig. 6a,b,d,e), with non-significant median changes of 3% and 11% overall, as compared to average reported increases for the same regions resulting from early-life stress of 95%^32–34^ and 135%^29, 30, 47^, respectively (Fig. 6c,f).

## Discussion

Our findings establish that DCCD leads to lasting changes in somatic development and adult behavior. Additionally, these effects depend on the developmental life stage at which disruption occurs. *In utero* disruptions induce more pronounced behavioral changes in adult offspring, regardless of neonatal or maternal condition. In contrast, adult body mass is influenced more by neonatal than *in utero* condition, suggesting actions of DCCD on multiple targets across development. Notably, we did not observe learning deficits in DCCD offspring, consistent with some earlier reports e.g., ref. 14. Our results establish that early life circadian disruption exerts life-stage-dependent effects, and that these effects are additive, suggesting that maternal and early life circadian rhythms play complex organizational roles in normal development.

Many types of learning show circadian modulation^49^. In the present study, behavioral testing was conducted during the day, which may affect how the mice responded to the stress of novel tests^50^. Although unlikely, it is possible that testing at this time masks subtle DCCD-induced learning deficits and testing in the early dark phase might permit unmasking^49^. Other studies have found learning deficits following gestation under constant light^15, 16^. In these cases, testing was conducted at variable times of day. Together, these findings suggest that the nature of the circadian disruption applied developmentally, and the time of day of testing, may influence those deficits that manifest.

Given that circadian disruptions can activate the stress axis, we speculated that ELS mechanisms may contribute to any observed changes. To explore this possibility, we examined markers of ELS in dams and offspring, but detected no signs of stress in any of the markers in any condition. DCCD dams did not have smaller or underweight litters and did not cannibalize their pups at an increased rate. Furthermore, CC and SS offspring exhibited levels of methylation of BDNF IV and GR 1_7_ comparable to control mice, and control values in the literature^29–32, 34, 47, 51, 52^. Specifically, SS shows non-significant 3% and 11% increases from CC, respectively, as compared to 95% and 135% increases seen with ELS for the same regions^29–32, 34, 47, 51, 52^. The failure to implicate ELS mechanisms, taken together with a limited role of melatonin in this strain of mice, points to a unique mechanism by which DCCD negatively affects offspring growth and adult behavior.

The present findings also indicate that both *in utero* and early life development are uniquely vulnerable, with different downstream consequences. Specifically, disruptions *in utero* lead to markedly modified behavioral phenotypes that are not rescued by quality maternal care; by contrast, early postnatal circadian disruption induces nearly equivalent behavioral changes and a more marked body mass deficit. Differences in body mass do not appear to be due to differential maternal care as DCCD offspring diverge from control animals after weaning. The present study did not examine body composition or food intake, providing opportunity for future studies to determine specific systems impacted and potential changes in feeding and metabolism resulting from DCCD. The failure of stable maternal rhythms to rescue offspring outcomes are in contrast with both the ELS and maternal melatonin literature, in which neonatal interventions rescue circadian-^22, 53–55^ and ELS-induced effects in offspring^56^. In the present study, use of BALB/c mice minimizes the role of melatonin in all groups, suggesting that circadian disruption can negatively affect development independently of alterations in this hormone^26, 27^. Mothers communicate rhythmic information to developing pups through melatonin in their milk^20, 22^. Whether a dam producing robust melatonin rhythms would rescue the impact of prenatal DCCD is not clear. Our findings suggest that even when dams lack robust melatonin rhythms, DCCD still has a pronounced effect compared to controls. A more complete understanding is therefore needed of the mechanisms contributing to the effects of DCCD, both *in utero* and in postnatal development.

Endogenous circadian rhythms are not manifest until late in fetal development^57^ and developing offspring rely on metabolic and hormonal information communicated from mother to fetuses^17, 20, 58^. Misalignment of these cues may disrupt the organization of pup physiology. As fetuses develop, they become sensitive to light cues penetrating through the mother’s abdomen^59^. Thus, pups may not only experience circadian disruptions through mistimed maternal signals, but also through their own internal misalignment to the light:dark environment. Importantly, temporal structure governing behavior in the mother, and likely in the fetus, is provided by ultradian (rhythms < 24 h) as well as circadian mechanisms. Several physiological systems exhibit ultradian rhythms (URs) in the 1–3 h range, including the HPG axis^60–65^, the HPA axis^66–72^, and the suprachiasmatic nucleus^73, 74^ – the principal orchestrating brain area for circadian rhythms. The central dopaminergic axis exhibits URs in the 1–3 h range^75^, and may influence CBT as well through modulation of activity and appetitive behaviors, such as eating and drinking (reviewed in ref. 76). We recently documented that maternal UR power early in pregnancy is predictive of pregnancy success^40^. Therefore, it is possible that URs play important roles in normal pup development. Fetal URs are understudied but do exist^77^ and may provide a substrate through which temporal disruptions manifest as disrupted signaling within the developing fetal physiological network^78^.

In summary, chronic circadian disruption during gestation affects maternal behavior and the behavioral phenotype of their offspring in adulthood, independent of ELS. These effects depend on the timing of developmental circadian disruption. Circadian disruptions are ubiquitous in modern society, affected by the modern lighting environment and negatively impacting health from *in utero* development through old age^79–81^. As of 2004, 12.6% of women over the age of 16 in the US work force were shift workers^82^. Given the lasting negative impacts on female reproductive health^83^, circadian disruption represents a highly relevant environmental threat to both women and their children. Our behavioral findings of hyperactivity and social avoidance are consistent with patterns seen in human adolescents. Sleep disruption in adolescents is associated with attention deficit hyperactivity disorder^84^ and autism spectrum disorder^85^. Diagnoses for both have been on the rise over the past several decades^86–88^. Our findings support the hypothesis that increased circadian disruption early in life, as in portable electronic devices and light pollution, may play a role in these increases in incidence. Large datasets of mothers and newborns (i.e., nuMoM2b^89^) are excellent potential sources of information about how DCCD may affect children. The present findings underscore the need for increased investigation of the effects of temporal disruption on development, both in model systems and in humans.

## Electronic supplementary material

Supplemental Figures 1 & 2

## References

[1] Smarr, B. L. & Kriegsfeld, L. J. In *AP*A Handbook *o*f Com*parative Psychology* (ed. Call, J.) 1, (Amer Psychological Assn, 2017).

[2] Evans JA, Davidson AJ (2013). Health consequences of circadian disruption in humans and animal models. Prog. Mol. Biol. Transl. Sci..

[3] Moore-Ede MC, Czeisler CA, Richardson GS (1983). Circadian timekeeping in health and disease. Part 1. Basic properties of circadian pacemakers. N. Engl. J. Med..

[4] Gibson EM, Wang C, Tjho S, Khattar N, Kriegsfeld LJ (2010). Experimental ‘jet lag’ inhibits adult neurogenesis and produces long-term cognitive deficits in female hamsters. PloS One.

[5] Roenneberg T, Merrow M (2016). The Circadian Clock and Human Health. Curr. Biol..

[6] Karatsoreos IN (2012). Effects of circadian disruption on mental and physical health. Curr. Neurol. Neurosci. Rep..

[7] Karatsoreos IN, Bhagat S, Bloss EB, Morrison JH, McEwen BS (2011). Disruption of circadian clocks has ramifications for metabolism, brain, and behavior. Proc. Natl. Acad. Sci. USA.

[8] Froy O (2011). Circadian Rhythms, Aging, and Life Span in Mammals. Physiology.

[9] Bronson SL, Bale TL (2016). The Placenta as a Mediator of Stress Effects on Neurodevelopmental Reprogramming. Neuropsychopharmacol. Off. Publ. Am. Coll. Neuropsychopharmacol..

[10] Bolton JL, Bilbo SD (2014). Developmental programming of brain and behavior by perinatal diet: focus on inflammatory mechanisms. Dialogues Clin. Neurosci..

[11] Leith Sly J, Carpenter DO (2012). Special vulnerability of children to environmental exposures. Rev. Environ. Health.

[12] Connors SL (2008). Fetal mechanisms in neurodevelopmental disorders. Pediatr. Neurol..

[13] Spichiger C (2015). Gestation under chronic constant light leads to extensive gene expression changes in the fetal rat liver. Physiol. Genomics.

[14] Roman E, Karlsson O (2013). Increased anxiety-like behavior but no cognitive impairments in adult rats exposed to constant light conditions during perinatal development. Ups. J. Med. Sci..

[15] Vilches N (2014). Gestational chronodisruption impairs hippocampal expression of NMDA receptor subunits Grin1b/Grin3a and spatial memory in the adult offspring. PloS One.

[16] Fujioka A (2011). Effects of a constant light environment on hippocampal neurogenesis and memory in mice. Neurosci. Lett..

[17] Torres-Farfan C (2004). Maternal melatonin selectively inhibits cortisol production in the primate fetal adrenal gland. J. Physiol..

[18] Varcoe TJ, Wight N, Voultsios A, Salkeld MD, Kennaway DJ (2011). Chronic phase shifts of the photoperiod throughout pregnancy programs glucose intolerance and insulin resistance in the rat. PloS One.

[19] Varcoe TJ (2013). Characterisation of the maternal response to chronic phase shifts during gestation in the rat: implications for fetal metabolic programming. PloS One.

[20] Illnerová H, Buresová M, Presl J (1993). Melatonin rhythm in human milk. J. Clin. Endocrinol. Metab..

[21] Nowak R, Young IR, McMillen IC (1990). Emergence of the diurnal rhythm in plasma melatonin concentrations in newborn lambs delivered to intact or pinealectomized ewes. J. Endocrinol..

[22] Cohen Engler A, Hadash A, Shehadeh N (2012). & Pillar, G. Breastfeeding may improve nocturnal sleep and reduce infantile colic: potential role of breast milk melatonin. Eur. J. Pediatr..

[23] Okatani Y (1998). Maternal-fetal transfer of melatonin in pregnant women near term. J. Pineal Res..

[24] Deguchi T (1975). Ontogenesis of a biological clock for serotonin:acetyl coenzyme A N-acetyltransferase in pineal gland of rat. Proc. Natl. Acad. Sci. USA.

[25] Kennaway DJ, Stamp GE, Goble FC (1992). Development of melatonin production in infants and the impact of prematurity. J. Clin. Endocrinol. Metab..

[26] Vivien-Roels B (1998). Daily Variations in Pineal Melatonin Concentrations in Inbred and Outbred Mice. J. Biol. Rhythms.

[27] Kennaway DJ, Voultsios A, Varcoe TJ, Moyer RW (2002). Melatonin in mice: rhythms, response to light, adrenergic stimulation, and metabolism. Am. J. Physiol. Regul. Integr. Comp. Physiol.

[28] McEwen BS, Karatsoreos IN (2015). Sleep Deprivation and Circadian Disruption: Stress, Allostasis, and Allostatic Load. Sleep Med. Clin..

[29] Weaver ICG (2004). Epigenetic programming by maternal behavior. Nat. Neurosci..

[30] Palma-Gudiel H, Córdova-Palomera A, Leza JC, Fañanás L (2015). Glucocorticoid receptor gene (NR3C1) methylation processes as mediators of early adversity in stress-related disorders causality: A critical review. Neurosci. Biobehav. Rev..

[31] Romens SE, McDonald J, Svaren J, Pollak SD (2015). Associations Between Early Life Stress and Gene Methylation in Children. Child Dev..

[32] Roth TL, Lubin FD, Funk AJ, Sweatt JD (2009). Lasting epigenetic influence of early-life adversity on the BDNF gene. Biol. Psychiatry.

[33] Unternaehrer E (2015). Childhood maternal care is associated with DNA methylation of the genes for brain-derived neurotrophic factor (BDNF) and oxytocin receptor (OXTR) in peripheral blood cells in adult men and women. Stress Amst. Neth..

[34] Blaze J, Asok A, Roth TL (2015). Long-term effects of early-life caregiving experiences on brain-derived neurotrophic factor histone acetylation in the adult rat mPFC. Stress Amst. Neth..

[35] Diego MA (2006). Maternal psychological distress, prenatal cortisol, and fetal weight. Psychosom. Med..

[36] Cheong, J. N. *et al*. Sex-specific metabolic outcomes in offspring of female rats born small or exposed to stress during pregnancy. *Endocrinology* en. 2016–1335 (2016). 10.1210/en.2016-1335 27571133

[37] Geraghty, A. C. *et al*. Knockdown of hypothalamic RFRP3 prevents chronic stress-induced infertility and embryo resorption. *eLife* **4**. 10.7554/eLife.04316 PMC4289855 25581095

[38] Busnel RG, Lehmann A (1977). Acoustic signals in mouse maternal behavior: retrieving and cannibalism. Z. Für Tierpsychol..

[39] Bellisario V (2015). Maternal high-fat diet acts as a stressor increasing maternal glucocorticoids’ signaling to the fetus and disrupting maternal behavior and brain activation in C57BL/6J mice. Psychoneuroendocrinology.

[40] Smarr BL, Zucker I, Kriegsfeld LJ (2016). Detection of Successful and Unsuccessful Pregnancies in Mice within Hours of Pairing through Frequency Analysis of High Temporal Resolution Core Body Temperature Data. PLOS ONE.

[41] Lalonde R, Strazielle C (2008). Relations between open-field, elevated plus-maze, and emergence tests as displayed by C57/BL6J and BALB/c mice. J. Neurosci. Methods.

[42] Han S (2012). Autistic-like behaviour in Scn1a ^+^/_−_ mice and rescue by enhanced GABA-mediated neurotransmission. Nature.

[43] Antunes M, Biala G (2012). The novel object recognition memory: neurobiology, test procedure, and its modifications. Cogn. Process.

[44] Braithwaite EC, Kundakovic M, Ramchandani PG, Murphy SE, Champagne FA (2015). Maternal prenatal depressive symptoms predict infant NR3C1 1F and BDNF IV DNA methylation. Epigenetics.

[45] Appleton AA, Lester BM, Armstrong DA, Lesseur C, Marsit CJ (2015). Examining the joint contribution of placental NR3C1 and HSD11B2 methylation for infant neurobehavior. Psychoneuroendocrinology.

[46] Jiang M (2009). Rapid quantification of DNA methylation by measuring relative peak heights in direct bisulfite-PCR sequencing traces. Lab. Invest..

[47] McGowan PO (2009). Epigenetic regulation of the glucocorticoid receptor in human brain associates with childhood abuse. Nat. Neurosci..

[48] Rokach, L. & Maimon, O. In *Data Mining and Knowledge* Discovery *Handbook* (eds Maimon, O. & Rokach, L.) 321–352 (Springer US, 2005).

[49] Smarr BL, Jennings KJ, Driscoll JR, Kriegsfeld LJ (2014). A time to remember: the role of circadian clocks in learning and memory. Behav. Neurosci..

[50] Koch CE, Leinweber B, Drengberg BC, Blaum C, Oster H (2016). Interaction between circadian rhythms and stress. Neurobiol. Stress.

[51] Perroud N (2011). Increased methylation of glucocorticoid receptor gene (NR3C1) in adults with a history of childhood maltreatment: a link with the severity and type of trauma. Transl. Psychiatry.

[52] Dong E (2015). BDNF epigenetic modifications associated with schizophrenia-like phenotype induced by prenatal stress in mice. Biol. Psychiatry.

[53] Lee TM, Spears N, Tuthill CR, Zucker I (1989). Maternal melatonin treatment influences rates of neonatal development of meadow vole pups. Biol. Reprod.

[54] Cisternas CD, Compagnucci MV, Conti NR, Ponce RH, Vermouth NT (2010). Protective effect of maternal prenatal melatonin administration on rat pups born to mothers submitted to constant light during gestation. Braz. J. Med. Biol. Res. Rev. Bras. Pesqui. Medicas E Biol..

[55] Grosse J, Velickovic A, Davis FC (1996). Entrainment of Syrian hamster circadian activity rhythms by neonatal melatonin injections. Am. J. Physiol. - Regul. Integr. Comp. Physiol..

[56] Francis D, Diorio J, Liu D, Meaney MJ (1999). Nongenomic Transmission Across Generations of Maternal Behavior and Stress Responses in the Rat. Science.

[57] Landgraf D, Koch CE, Oster H (2014). Embryonic development of circadian clocks in the mammalian suprachiasmatic nuclei. Front. Neuroanat..

[58] Thomas KA, Burr RL, Spieker S, Lee J, Chen J (2014). Mother-infant circadian rhythm: development of individual patterns and dyadic synchrony. Early Hum. Dev..

[59] Rao S (2013). A direct and melanopsin-dependent fetal light response regulates mouse eye development. Nature.

[60] Sanchez-Alavez M, Alboni S, Conti B (2011). Sex- and age-specific differences in core body temperature of C57Bl/6 mice. Age Dordr. Neth..

[61] Mauvais-Jarvis F (2011). Estrogen and androgen receptors: regulators of fuel homeostasis and emerging targets for diabetes and obesity. Trends Endocrinol. Metab. TEM.

[62] Marrone BL, Thomas Gentry R, Wade GN (1976). Gonadal hormones and body temperature in rats: Effects of estrous cycles, castration and steroid replacement. Physiol. Behav..

[63] Rance NE, Dacks PA, Mittelman-Smith MA, Romanovsky AA, Krajewski-Hall SJ (2013). Modulation of body temperature and LH secretion by hypothalamic KNDy (kisspeptin, neurokinin B and dynorphin) neurons: a novel hypothesis on the mechanism of hot flushes. Front. Neuroendocrinol..

[64] de la Iglesia HO, Schwartz WJ (2006). Minireview: timely ovulation: circadian regulation of the female hypothalamo-pituitary-gonadal axis. Endocrinology.

[65] Wang ZY, Cable EJ, Zucker I, Prendergast BJ (2014). Pregnancy-induced changes in ultradian rhythms persist in circadian arrhythmic Siberian hamsters. Horm. Behav..

[66] Smarr, B., Burnett, D., Mesri, S., Pister, K. & Kriegsfeld, L. A Wearable Sensor System with Circadian Rhythm Stability Estimation for Prototyping Biomedical Studies. *IEEE Trans. Affect. Comput*. PP, 1–1 (2015).

[67] Fulkerson WJ, Tang BY (1979). Ultradian and circadian rhythms in the plasma concentration of cortisol in sheep. J. Endocrinol..

[68] Young EA, Abelson J, Lightman SL (2004). Cortisol pulsatility and its role in stress regulation and health. Front. Neuroendocrinol..

[69] Qian X, Droste SK, Lightman SL, Reul JMHM, Linthorst ACE (2012). Circadian and ultradian rhythms of free glucocorticoid hormone are highly synchronized between the blood, the subcutaneous tissue, and the brain. Endocrinology.

[70] Plat L (1996). Effects of morning cortisol elevation on insulin secretion and glucose regulation in humans. Am. J. Physiol..

[71] Sanchez-Alavez M (2010). Insulin causes hyperthermia by direct inhibition of warm-sensitive neurons. Diabetes.

[72] Lambillotte C, Gilon P, Henquin JC (1997). Direct glucocorticoid inhibition of insulin secretion. An in vitro study of dexamethasone effects in mouse islets. J. Clin. Invest..

[73] Ono D, Honma K, Honma S (2015). Circadian and ultradian rhythms of clock gene expression in the suprachiasmatic nucleus of freely moving mice. Sci. Rep..

[74] Weinert D, Waterhouse J (2007). The circadian rhythm of core temperature: Effects of physical activity and aging. Physiol. Behav..

[75] Blum, I. D. *et al*. A highly tunable dopaminergic oscillator generates ultradian rhythms of behavioral arousal. *eLife* **3** (2014). 10.7554/eLife.05105 PMC4337656 25546305

[76] Prendergast BJ, Zucker I (2016). Ultradian rhythms in mammalian physiology and behavior. Curr. Opin. Neurobiol..

[77] Pillai M, James DK, Parker M (1992). The development of ultradian rhythms in the human fetus. Am. J. Obstet. Gynecol..

[78] Feldman R (2007). Parent-infant synchrony and the construction of shared timing; physiological precursors, developmental outcomes, and risk conditions. J. Child Psychol. Psychiatry.

[79] Borniger JC, McHenry ZD, Abi Salloum BA, Nelson RJ (2014). Exposure to dim light at night during early development increases adult anxiety-like responses. Physiol. Behav..

[80] Cissé YM, Peng J, Nelson RJ (2016). Dim light at night prior to adolescence increases adult anxiety-like behaviors. Chronobiol. Int..

[81] Hofman MA, Swaab DF (1994). Alterations in circadian rhythmicity of the vasopressin-producing neurons of the human suprachiasmatic nucleus (SCN) with aging. Brain Res..

[82] McMenamin, T. M. A time to work: Recent trends in shift work and flexible schedules. *Mon. Labor Rev*. (2007).

[83] Gamble, K. L., Resuehr, D. & Johnson, C. H. Shift Work and Circadian Dysregulation of Reproduction. *Front. Endocrinol*. **4** (2013). 10.3389/fendo.2013.00092 PMC3736045 23966978

[84] Lunsford-Avery JR, Krystal AD, Kollins SH (2016). Sleep disturbances in adolescents with ADHD: A systematic review and framework for future research. Clin. Psychol. Rev..

[85] Cohen, S., Conduit, R., Lockley, S. W., Rajaratnam, S. M. & Cornish, K. M. The relationship between sleep and behavior in autism spectrum disorder (ASD): a review. *J. Neurodev. Disord*. **6** (2014). 10.1186/1866-1955-6-44 PMC4271434 25530819

[86] Olfson M, Gameroff MJ, Marcus SC, Jensen PS (2003). National Trends in the Treatment of Attention Deficit Hyperactivity Disorder. Am. J. Psychiatry.

[87] Giacobini, M., Medin, E., Ahnemark, E., Russo, L. J. & Carlqvist, P. Prevalence, Patient Characteristics, and Pharmacological Treatment of Children, Adolescents, and Adults Diagnosed With ADHD in Sweden. *J. Atten. Disord*. 1087054714554617 (2014). 10.1177/1087054714554617 25376193

[88] Mandell DS, Thompson WW, Weintraub ES, DeStefano F, Blank MB (2005). Trends in Diagnosis Rates for Autism and ADHD at Hospital Discharge in the Context of Other Psychiatric Diagnoses. Psychiatr. Serv..

[89] Haas DM (2015). A description of the methods of the Nulliparous Pregnancy Outcomes Study: monitoring mothers-to-be (nuMoM2b). Am. J. Obstet. Gynecol..

