# Supplementary material for: Maternal and Early-Life Circadian Disruption Have Long-Lasting Negative Consequences on Offspring Development and Adult Behavior in Mice
Source: Sci Rep. 2017 Jun 12;7:3326. doi: 10.1038/s41598-017-03406-4 (PMC5468226; doi:10.1038/s41598-017-03406-4)
Supplement: Supplementary file 1 — Supplemental Figures 1 & 2 [file 41598_2017_3406_MOESM1_ESM.pdf]

Title: **Maternal and Early-Life Circadian Disruption Have Long-Lasting Negative Consequences on Offspring Development and Adult Behavior in Mice**

Short title: **Life-Long Role for Daily Timing in Development**

Authors:
Benjamin L. Smarr, Azure D. Grant, Luz Perez, Irving Zucker, & Lance J. Kriegsfeld

Supplemental Figures

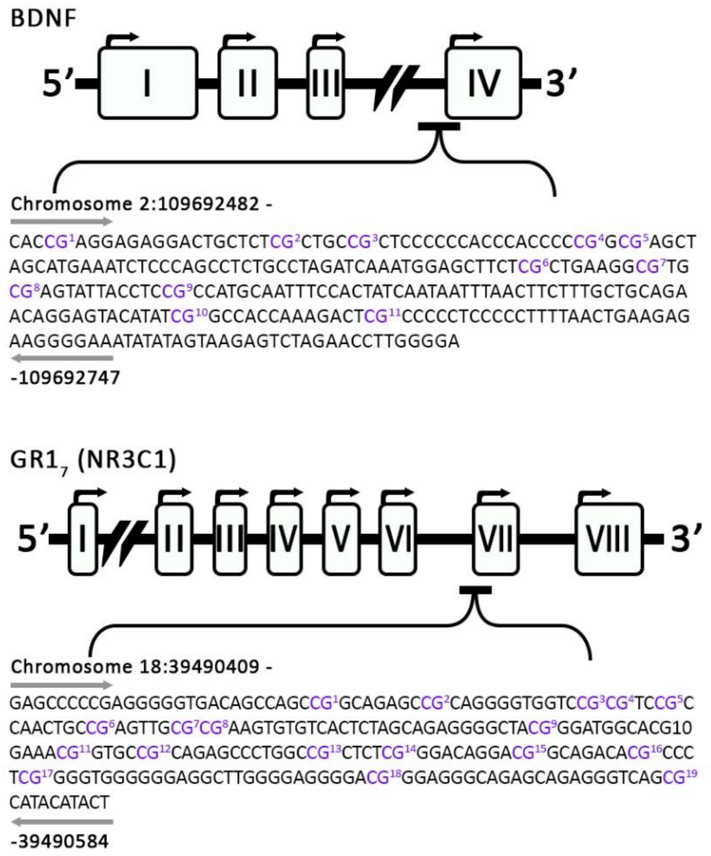

Supplementary Figure S1. Methylation maps. Genes are depicted with coding regions labelled in roman numerals. Arrows indicate promoter regions. Amplicons are represented by black bars beneath the region described in[32–34] as exon IV promoter region for BDNF (A). The NR3C1 amplicon in the promoter region of exon 1[7] is represented similarly[29,30,45]. Chromosomal location according to the mouse genome assembly GRCm38.p4 (Jan 2012) is shown at the beginning and end of each sequence. CpGs captured are depicted in red and are numbered sequentially.

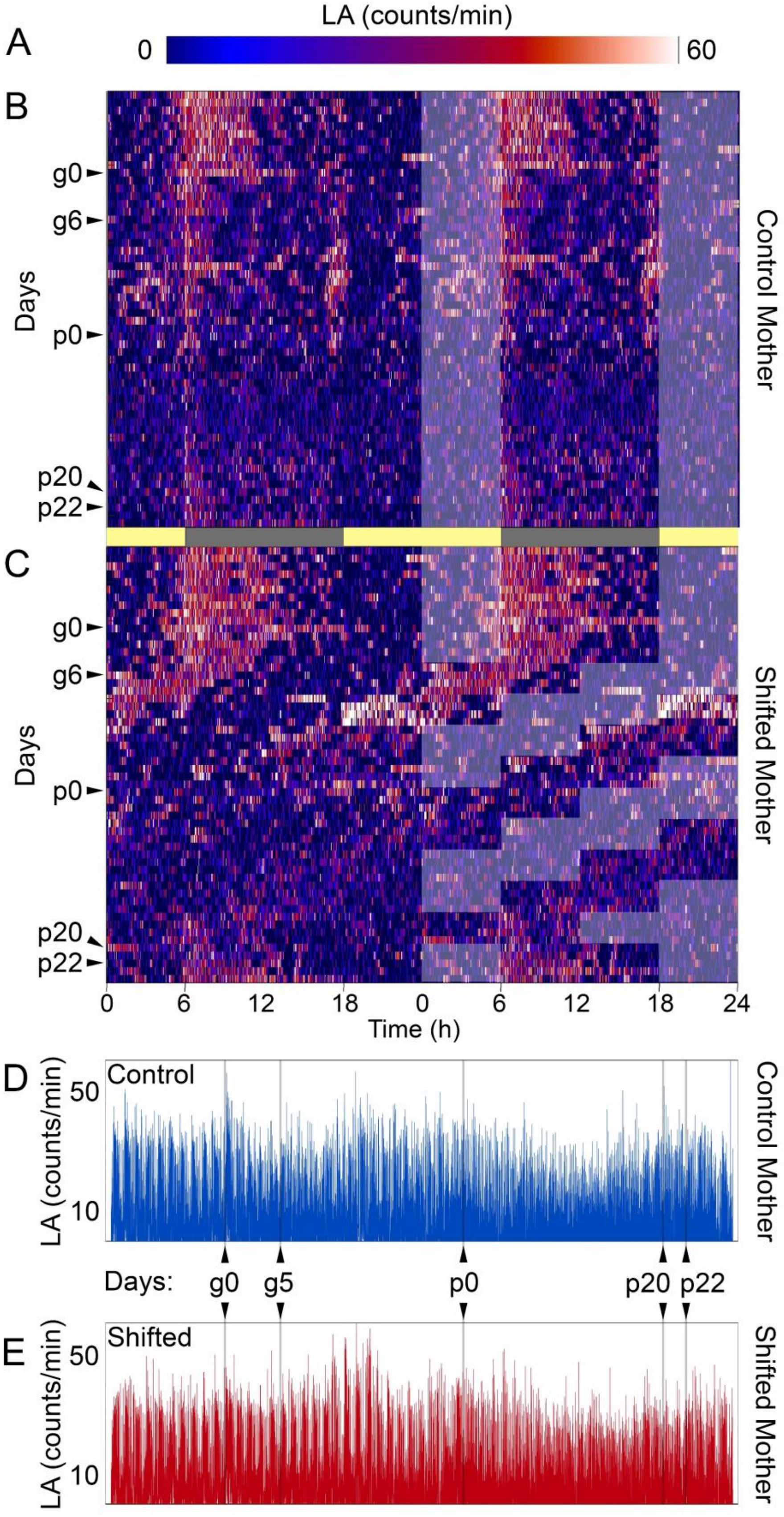

A
LA (counts/min)
0
60
B
g0
g6
Days
p0
p20
p22
Control Mother
C
g0
g6
Days
p0
p20
p22
Shifted Mother
0 6 12 18 0 6 12 18 24
Time (h)
D
Control
LA (counts/min)
50
10
Control Mother
Days:
g0
g5
p0
p20
p22
E
Shifted
LA (counts/min)
50
10
Shifted Mother

17

Supplemental Figure S2. Maternal locomotor activity differs from CBT, but also shows changes due to advances in LD schedule. Color maps of intensity (A) reveal stable daily rhythms of locomotor activity (LA) in sensor-implanted dams from control (B) and shifting (C) conditions before the onset of DCCD. Double-plotted (i.e., 48 h) actograms, with light-phase overlain on the right half. After onset of DCCD, shifting dams show expected advances in their LA rhythms in response to advances of the LD cycle, which overall give shifting dams 2 additional daily cycles during their pregnancies (48 h accumulated advance). LA reveals less stereotyped change across pregnancies (D, E) than does CBT (Fig. 2).
